# Dysfunction of cAMP–Protein Kinase A–Calcium Signaling Axis in Striatal Medium Spiny Neurons: A Role in Schizophrenia and Huntington’s Disease Neuropathology

**DOI:** 10.1016/j.bpsgos.2022.03.010

**Published:** 2022-04-04

**Authors:** Marija Fjodorova, Zoe Noakes, Daniel C. De La Fuente, Adam C. Errington, Meng Li

**Affiliations:** a Neuroscience and Mental Health Research Institute, School of Medicine, Cardiff University, Cardiff, United Kingdom; b Division of Neuroscience, School of Bioscience, Cardiff University, Cardiff, United Kingdom

**Keywords:** BCL11B, Calcium signaling, Dopaminergic synapse, Glutamatergic synapse, Medium spiny neuron, Psychiatric disorder

## Abstract

**Background:**

Striatal medium spiny neurons (MSNs) are preferentially lost in Huntington’s disease. Genomic studies also implicate a direct role for MSNs in schizophrenia, a psychiatric disorder known to involve cortical neuron dysfunction. It remains unknown whether the two diseases share similar MSN pathogenesis or if neuronal deficits can be attributed to cell type–dependent biological pathways. Transcription factor BCL11B, which is expressed by all MSNs and deep layer cortical neurons, was recently proposed to drive selective neurodegeneration in Huntington’s disease and identified as a candidate risk gene in schizophrenia.

**Methods:**

Using human stem cell–derived neurons lacking BCL11B as a model, we investigated cellular pathology in MSNs and cortical neurons in the context of these disorders. Integrative analyses between differentially expressed transcripts and published genome-wide association study datasets identified cell type–specific disease-related phenotypes.

**Results:**

We uncover a role for BCL11B in calcium homeostasis in both neuronal types, while deficits in mitochondrial function and PKA (protein kinase A)–dependent calcium transients are detected only in MSNs. Moreover, BCL11B-deficient MSNs display abnormal responses to glutamate and fail to integrate dopaminergic and glutamatergic stimulation, a key feature of striatal neurons in vivo. Gene enrichment analysis reveals overrepresentation of disorder risk genes among BCL11B-regulated pathways, primarily relating to cAMP-PKA-calcium signaling axis and synaptic signaling.

**Conclusions:**

Our study indicates that Huntington’s disease and schizophrenia are likely to share neuronal pathophysiology where dysregulation of intracellular calcium homeostasis is found in both striatal and cortical neurons. In contrast, reduction in PKA signaling and abnormal dopamine/glutamate receptor signaling is largely specific to MSNs.

Inhibitory GABA (gamma-aminobutyric acid)–releasing medium spiny neurons (MSNs) are the principal projection neurons of the basal ganglia, receiving inputs from both cortical glutamatergic neurons and midbrain dopaminergic (mDA) neurons. MSNs are critically involved in a variety of essential functions including voluntary motor control, habit learning, and reward processing, and dysfunction followed by loss of this neuron population underlies Huntington’s disease (HD). A growing body of evidence identifies loss of function of transcription factor BCL11B (also known as CTIP2) as a driving force behind selective neuron degeneration in HD.

*BCL11B* is expressed by all MSNs and is required for transcriptional regulation of striatal genes, patch-matrix organization, spatial learning, and working memory (1, 2, 3, 4). *BCL11B* is also highly expressed by cortical layer V/VI neurons, where it plays a role in corticospinal motor neuron fate specification and axon development (5,6). BCL11B protein level is reduced in both human and rodent mutant huntingtin (mHTT)–expressing cells, resulting in mitochondrial deficits prior to the onset of MSN death (7, 8, 9, 10). We have recently demonstrated that human BCL11B-deficient and HD MSNs share dysregulated gene expression and present with deficits in signature striatal protein phosphorylation, DARPP32, and GLUR1 (11). In addition to the striatum, *BCL11B*-expressing neurons in other brain regions are affected in the later stages of HD, such as the cortex, hippocampus, and hypothalamus (8). Thus, these findings point to an important role for BCL11B in HD pathogenesis.

Loss-of-function mutations in the *BCL11B* gene have also been identified to cause immunodeficiency and neurodevelopmental delay with speech impairment and intellectual disability (12). Furthermore, several studies have recently demonstrated genome-wide significant enrichment of polymorphisms increasing risk for schizophrenia (SCZ) in the *BCL11B* gene (13, 14, 15, 16, 17). It has been widely accepted that pathogenesis in neurodevelopmental and psychiatric disorders is driven by cortical interneuron and glutamatergic neuron dysfunction (18), but novel evidence suggests that MSNs also play an important distinct role. Several gene enrichment studies integrating single-cell transcriptomics and large psychiatric genome-wide association study datasets revealed that psychiatric risk variants were highly enriched in genes expressed by MSNs that differ from genes expressed by cortical neurons (17,19, 20, 21). Considering that polymorphisms affect both pan-neuronal and subtype-specific neuronal genes, it is important and necessary to investigate whether resulting cellular pathology is distinct or shared between striatal and cortical cell populations. It also remains to be determined as to what extent HD and SCZ neuropathology may overlap due to disrupted function of BCL11B, and if the same or different biological pathways are at play in these conditions. Together, these findings lead to the hypothesis that BCL11B regulates important signaling processes in MSNs and cortical neurons, whether shared or distinct, that may be particularly vulnerable to psychiatric disorder risk variants and HD pathogenesis.

Using BCL11B-deficient human embryonic stem cells as a model to address the above questions, we demonstrate here a role for BCL11B in mitochondrial function, calcium signaling, and dopamine/glutamate signal processing predominantly in MSNs, with much milder deficits observed in cortical neurons. Our study reveals significant enrichment of psychiatric disorder risk genes in BCL11B-regulated signaling, including cAMP-dependent protein kinase A (PKA) and DARPP32 signaling, specifically in MSNs, and calcium and glutamatergic synaptic signaling in both neuronal types. Similar biological pathways are identified in HD, suggesting a shared role for BCL11B in the pathophysiology of HD and SCZ. We therefore prioritize promising targets for further mechanistic investigations and development of new therapeutics in these conditions.

## Methods and Materials

### Cell Culture and Neuronal Differentiation

**Figure 1:**
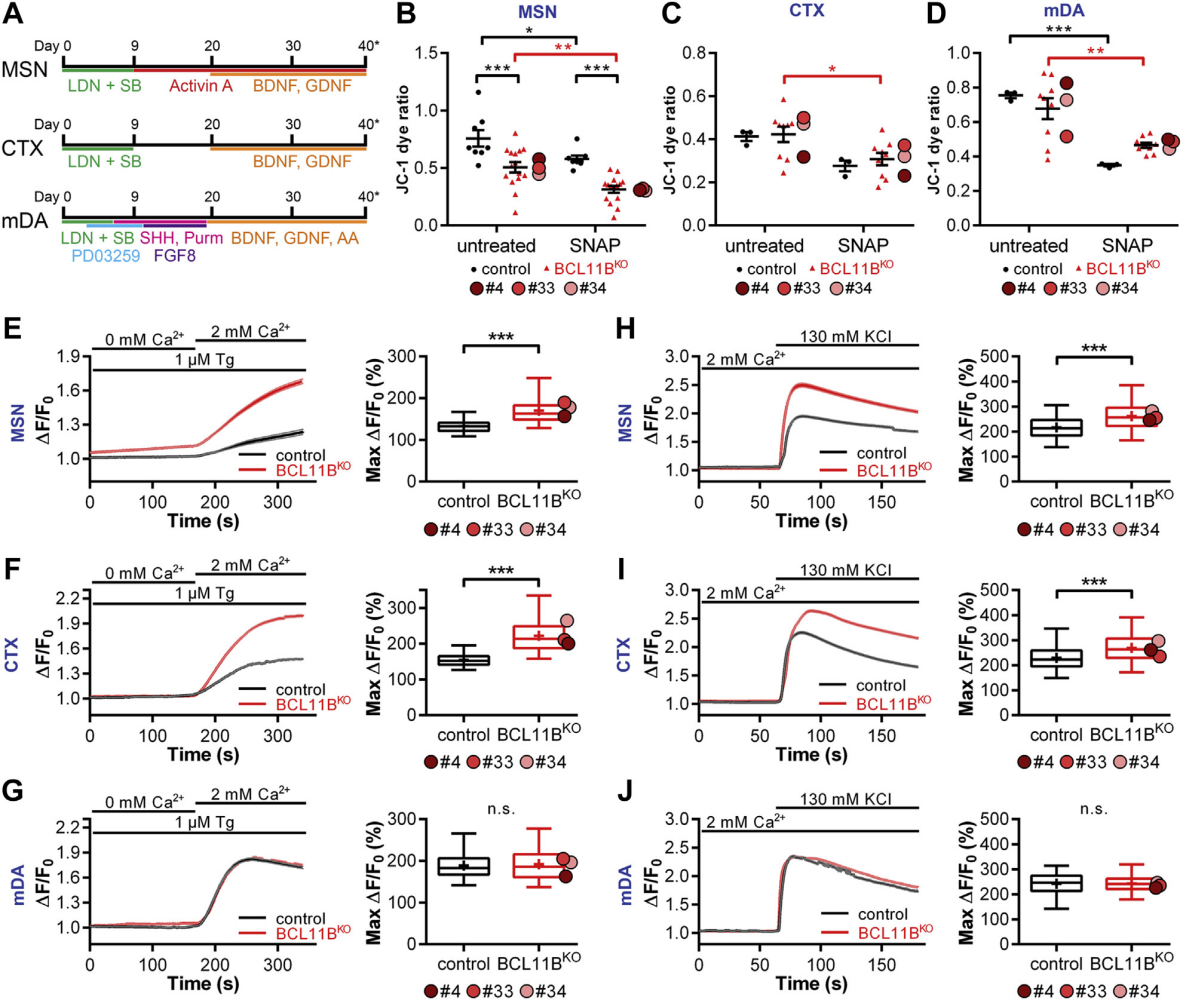
BCL11B^KO^ MSNs and CTX neurons manifest signs of mitochondrial dysfunction and abnormal intracellular Ca^2+^ regulation. **(A)** Schematic of MSN, CTX, and mDA neuron differentiation protocols. **(B–D)** Mitochondrial membrane potential as measured by JC-1 dye ratio at rest and after 24 hours treatment with 1000 μM SNAP in **(B)** MSNs, **(C)** CTX neurons, and **(D)** mDA neurons at 40 days of differentiation (two-way analysis of variance with post hoc Bonferroni test; MSN: genotype, *F*_1,42_ = 30.552, *p* = 1.9 × 10^−6^; treatment, *F*_1,42_ = 15.601, *p* = 2.9 × 10^−4^; from left, in black: ∗∗∗*p* = 4.6 × 10^−4^, 2.4 × 10^−4^; ∗*p* = .024; in red: ∗∗*p* = .001; CTX: treatment, *F*_1,20_ = 8.863, *p* = .007; in red: ∗*p* = .013; mDA: treatment, *F*_1,20_ = 29.941, *p* = 2.3 × 10^−5^; in black: ∗∗∗*p* = 4.9 × 10^−4^; in red: ∗∗*p* = .001). **(E–G)** Significantly increased store-operated channel-mediated Ca^2+^ entry in response to thapsigargin) is observed in BCL11B^KO^ vs. control MSNs and CTX neurons but not mDA neurons, as demonstrated by Δ*F/F*_*0*_ traces (left) and quantification of maximum Δ*F/F*_*0*_ (right) (Mann-Whitney *U* test; MSN: *U* = 63,008.5, ∗∗∗*p* = 2.4 × 10^−116^; CTX: *U* = 33,264, ∗∗∗*p* = 5.8 × 10^−192^). **(H–J)** Greater Ca^2+^ signals are induced in BCL11B^KO^ vs. control MSNs and CTX neurons in response to external KCl, as demonstrated by Δ*F/F*_*0*_ traces (left) and quantification of maximum Δ*F/F*_*0*_ (right) (Mann-Whitney *U* test; MSN: *U* = 515,797, ∗∗∗*p* = 3.3 × 10^−88^; CTX: *U* = 344,257, ∗∗∗*p* = 1.5 × 10^−54^). All line graphs and dot plots depict mean ± SEM for each genotype. Box-and-whisker plots depict data for each genotype (center line, median; +, mean; box limits, upper and lower quartiles; whiskers, 2.5 and 97.5 percentiles). Means for individual clones are indicated by red-shaded circles next to BCL11B^KO^ data. See also Figures S1 and S2. BDNF, brain-derived neurotrophic factor; CTX, cortical glutamatergic; KCI, potassium chloride; KO, knockout; LDN, LDN-193189; mDA, midbrain dopaminergic; MSNs, medium spiny neurons; n.s., not significant; SB, SB431542; SHH, sonic hedgehog; Tg, thapsigargin.

All experiments were performed in the HUES9 iCas9 hESC line and genome-edited derivatives lacking BCL11B protein (clones #4, #33, #34) (11). As in previous studies, BCL11B knockout (BCL11B^KO^) and control lines were reliably differentiated into MSN, cortical glutamatergic, and mDA neuronal subtypes using established protocols (Figure 1A; Figure S1) (22, 23, 24).

### Mitochondrial Assay

The cell-permeant mitochondrial membrane potential sensor JC-1 (Thermo Fisher Scientific) was added directly to live cells at a final concentration of 1 μg/mL for 30 minutes at 37 °C. Where indicated cells were preincubated with the nitric oxide donor SNAP (1000 μM; Tocris) for 24 hours. Samples were analyzed on a flow cytometer according to the manufacturer’s protocol.

### Live-Cell Calcium Imaging

Neuronal cultures were incubated with an intracellular Ca^2+^–sensitive probe Fluo-4-AM (Thermo Fisher Scientific) at a final concentration of 5 μM for 30 minutes at 37 °C. The solution was then replaced with prewarmed artificial cerebrospinal fluid (aCSF) solution (2 mM CaCl_2_, 142 mM NaCl, 2.5 mM KCl, 1 mM MgCl_2_, 10 mM HEPES, 30 mM D-glucose; all from Merck Sigma-Aldrich). Where indicated, aCSF was supplemented with one of the following: 1 μM thapsigargin, 10 μM roscovitine, 10 μM SKF-81297, 10 μM 8-bromo-cAMP (all from Tocris), 130 mM KCl, or 20 μM DL-glutamic acid (Merck Sigma-Aldrich). Subsequent data processing was performed using the FluoroSNNAP algorithm in MATLAB (version R2016a; The MathWorks, Inc.) according to the protocol (25).

### Electrophysiology

From 20 days of differentiation (days in vitro [DIV]), MSN progenitors were cultured in astrocyte-conditioned medium supplemented with maturation factors according to an established protocol (26). Whole-cell patch-clamp recordings were acquired from control and BCL11B^KO^ #4 MSNs at 40 DIV in aCSF at room temperature. SKF-81297 (10 μM) was added to aCSF where specified, while DL-glutamic acid (200 μM) was focally applied (30 ms) to the patched cell and the evoked current response recorded. Data were analyzed with Clampfit software (Molecular Devices) and exported to and plotted using Origin (OriginLab).

### RNA Sequencing Data Analysis

Paired-end sequencing was performed at Oxford Genomic Centre on an Illumina HiSeq 4000 (Illumina), and RNA sequencing data reported in this paper are available with the SRA accession numbers PRJNA474679 (https://www.ncbi.nlm.nih.gov/sra/PRJNA474679) and PRJNA767962 (https://www.ncbi.nlm.nih.gov/sra/PRJNA767962). The following R/Bioconductor packages and software were used to analyze differentially expressed genes and affected signaling pathways: DESeq2 (v.1.14.1) (27), clusterProfiler (v.3.12.14) (28), and Ingenuity Pathway Analysis (Qiagen).

### Statistical Analysis

For normally distributed data as determined by the Shapiro-Wilk test, we performed either two-tailed Student *t* test or two-way analysis of variance followed by post hoc Bonferroni correction for multiple comparisons. Alternatively, data were subjected to nonparametric Mann-Whitney *U* test or Kruskal-Wallis test followed by post hoc Bonferroni test.

## Results

### BCL11B-Deficient MSNs Display Mitochondrial Deficits

We showed previously that BCL11B^KO^ MSNs exhibited increased oxidative stress–dependent cell death (11). Because mitochondria are known to play an active role in this complex cascade of events in various models of neurodegenerative disorders (29), we set out to investigate whether loss of BCL11B would compromise mitochondrial health in MSNs, cortical neurons, and mDA neurons. Mitochondrial membrane potential sensor JC-1 was used to measure metabolic activity, a key indicator of mitochondrial health (Figure S2A) (30). Mitochondria in BCL11B^KO^ MSNs were found to be significantly more depolarized than in control cells at 40 DIV, indicating decreased metabolic activity in these neurons (Figure 1B).

To investigate whether this impairment in BCL11B^KO^ MSNs would render them more susceptible to oxidative stress, cells were treated with the nitric oxide donor SNAP, which was previously shown to generate reactive oxygen species (31). This induced a marked depolarization of the mitochondrial membrane in both groups, with a twofold greater amplitude in BCL11B^KO^ cultures (Figure 1B, Figure S2B). We ascertained that the observed mitochondrial deficit was BCL11B-dependent in MSNs by repeating this assay in striatal progenitors prior to BCL11B expression and detecting no deficit (Figure S2C).

We next investigated whether these mitochondrial deficits were specific to MSNs by performing the above experiment in cortical and mDA cultures. Although exposure to SNAP had a significant depolarizing effect in BCL11B^KO^ but not control cortical neurons after Bonferroni correction, there were no differences between the genotypes in either treatment group (Figure 1C). In addition, no differences were detected due to loss of BCL11B in mDA neurons, with both groups responding to SNAP similarly (Figure 1D). Taken together, our data suggest that BCL11B is required for regulation of mitochondrial membrane potential and has a protective role against oxidative stress, most prominently in MSNs and, to a lesser extent, in cortical neuron cultures.

### BCL11B^KO^ Neurons Present With Abnormal Intracellular Ca^2+^ Regulation

Mitochondrial dysfunction in neurons has previously been attributed to high intracellular Ca^2+^ levels, particularly in the context of neurologic disorders (32). Given the impairments in mitochondrial health in BCL11B^KO^ neurons, we hypothesized that loss of BCL11B would result in deficits in intracellular Ca^2+^ signaling and therefore performed calcium imaging in BCL11B^KO^ and control neuronal cultures at 50 DIV. To investigate the effect of BCL11B loss on the regulation of Ca^2+^ levels, intracellular Ca^2+^ was depleted using a Ca^2+^-free aCSF solution and store-operated channel-mediated Ca^2+^ entry was induced by application of the sarco/endoplasmic reticulum Ca^2+^ ATPase inhibitor thapsigargin (33,34). Significantly larger store-operated channel-mediated Ca^2+^ signals, suggesting greater Ca^2+^ influx, were detected in both BCL11B^KO^ MSNs and cortical, but not mDA, neurons compared with their respective control cells (Figure 1E–G). Consistent with this finding, markedly larger Ca^2+^ signals in both BCL11B^KO^ MSNs and cortical, but not mDA, neurons were also observed in response to depolarization of the plasma membrane by external potassium chloride (Figure 1H–J). These data indicate that BCL11B-dependent abnormal regulation of intracellular Ca^2+^ may underlie the depolarized mitochondrial membrane potential and increased vulnerability to oxidative stress in MSNs and cortical neurons described above.

### Signaling Deficits in Calcium Transients Are Rescued by PKA Activation in BCL11B^KO^ MSNs

**Figure 2:**
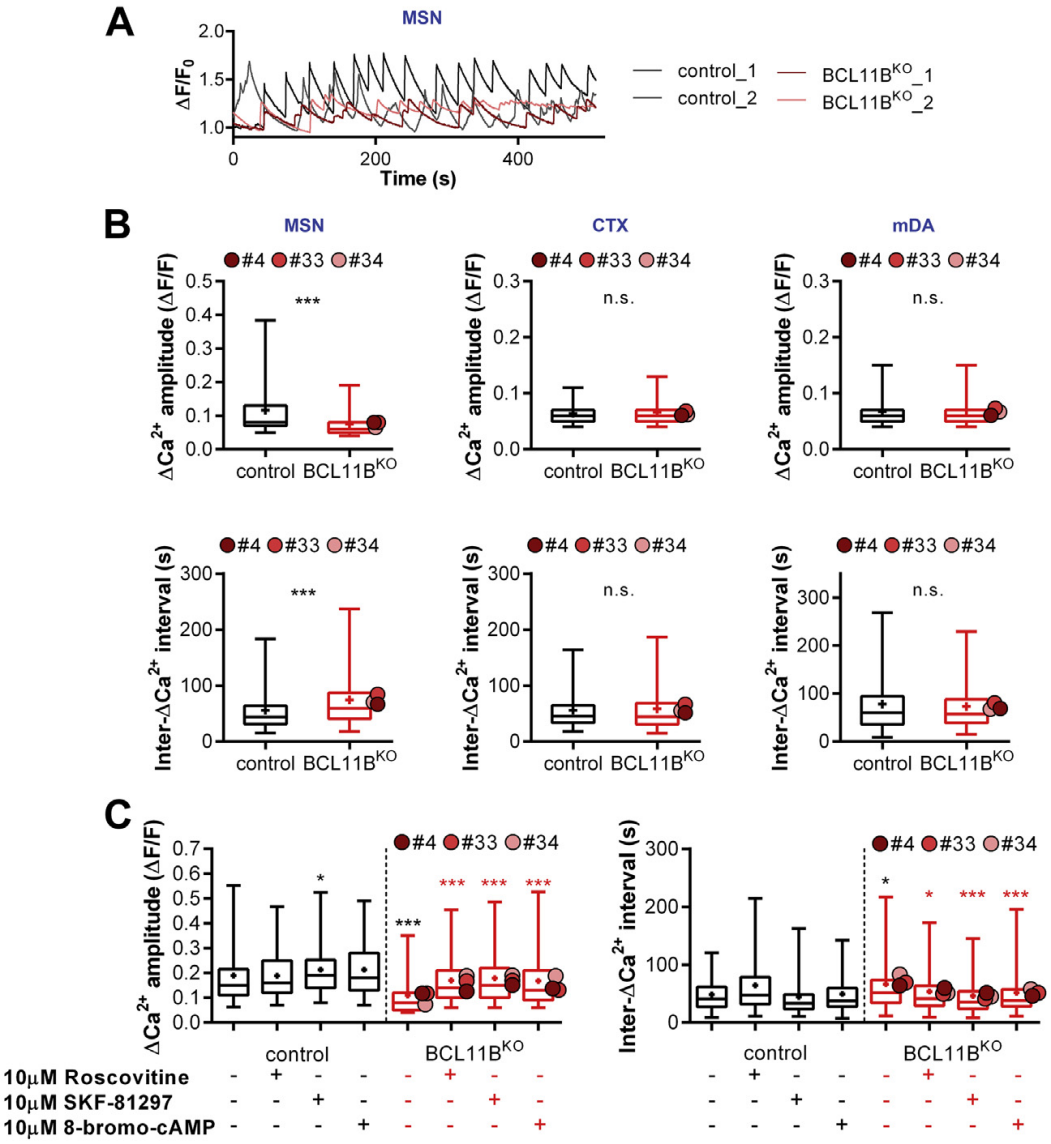
BCL11B^KO^–induced abnormal calcium transients are rescued by protein kinase A activation in MSNs. **(A)** Representative traces of spontaneous activity–evoked Ca^2+^ transients (ΔCa^2+^) in control and BCL11B^KO^ MSNs. **(B)** BCL11B-dependent reduced ΔCa^2+^ amplitudes and increased inter-ΔCa^2+^ intervals are present in MSNs but not CTX or mDA neurons (Mann-Whitney *U* test: MSN amplitude: *U* = 489,091.5, ∗∗∗*p* = 1.8 × 10^−85^; MSN interval: *U* = 610,109, ∗∗∗*p* = 3.4 × 10^−31^). **(C)** Lower ΔCa^2+^ amplitudes (left) and longer intervals (right) in BCL11B^KO^ vs. control MSNs are rescued in a protein kinase A–dependent manner with application of 10 μM roscovitine, 10 μM SKF-81927, or 10 μM 8-bromo-cAMP (Kruskal-Wallis test with post hoc Bonferroni test: amplitude: χ^2^_7_ = 275.877, *p* = 8.5 × 10^−56^; from left, in black: ∗*p* = .016, ∗∗∗*p* = 3.1 × 10^−^^24^ vs. untreated control; in red: ∗∗∗*p* = 2.5 × 10^−24^, 5.8 × 10^−25^, 3.7 × 10^−20^ vs. untreated BCL11B^KO^; ISI: χ^2^_7_ = 78.147, *p* = 3.3 × 10^−14^; from left, in black: ∗*p* = .013 vs. untreated control; in red: ∗*p* = .020, ∗∗∗*p* = 3.8 × 10^−9^, 1.02 × 10^−4^ vs. untreated BCL11B^KO^. Box-and-whisker plots depict data for each genotype (center line, median; +, mean; box limits, upper and lower quartiles; whiskers, 2.5 and 97.5 percentiles). Means for individual clones are indicated by red-shaded circles next to BCL11B^KO^ data. See also Figure S3. CTX, cortical glutamatergic; ISI, interspike interval; KO, knockout; mDA, midbrain dopamine; MSNs, medium spiny neurons; n.s., not significant.

Intracellular Ca^2+^ levels are also affected by Ca^2+^ influx and Ca^2+^ buffering during neuronal activity, which produces Ca^2+^ oscillations. Having established that BCL11B^KO^ MSNs and cortical neurons exhibit a notable deficit in the regulation of intracellular Ca^2+^ levels, we next examined spontaneous activity–evoked Ca^2+^ transients (ΔCa^2+^) in these cells (Figure S3A). In MSN cultures, smaller and less frequent ΔCa^2+^ were detected in BCL11B^KO^ neurons compared with control cells (Figure 2A). Indeed, this was reflected in a 36% decrease in ΔCa^2+^ amplitude and a 33% increase in inter-ΔCa^2+^ interval in BCL11B^KO^ MSNs (Figure 2B). This phenotype was specific to MSNs, because no deficits were detected in BCL11B^KO^ cortical and mDA neurons, either in ΔCa^2+^ amplitude or intervals (Figure 2B).

We next sought to gain mechanistic insight into the reduction in spontaneous ΔCa^2+^ in BCL11B^KO^ MSNs. Cross-talk between PKA and dopamine signaling pathways plays an important role in calcium signaling and DARPP32 phosphorylation in MSNs (35,36). Given reduced levels of DARPP32-Thr34 phosphorylation and PKA signaling in BCL11B^KO^ MSNs identified previously (11), we hypothesized that activating the PKA pathway would rescue ΔCa^2+^ deficits in these neurons. To this end, we compared the effects of drugs known to modulate PKA/DRD1-mediated calcium signaling in the striatum, including CDK5 inhibitor roscovitine, DRD1/DRD5 agonist SKF-81297, and cAMP analog 8-bromo-cAMP (35,37). All three treatments restored both the decreased ΔCa^2+^ amplitude and longer intervals in BCL11B^KO^ MSNs back to levels observed in control cells (Figure 2C, Figure S3B). Together, these findings suggest that loss of BCL11B disrupts PKA-dependent calcium signaling in MSNs, a deficit in spontaneous ΔCa^2+^ that is not observed in either cortical or mDA neurons.

### Abolished Dopaminergic Modulation of Excitatory Signaling and Impaired Glutamate-Evoked Responses in BCL11B^KO^ MSNs

**Figure 3:**
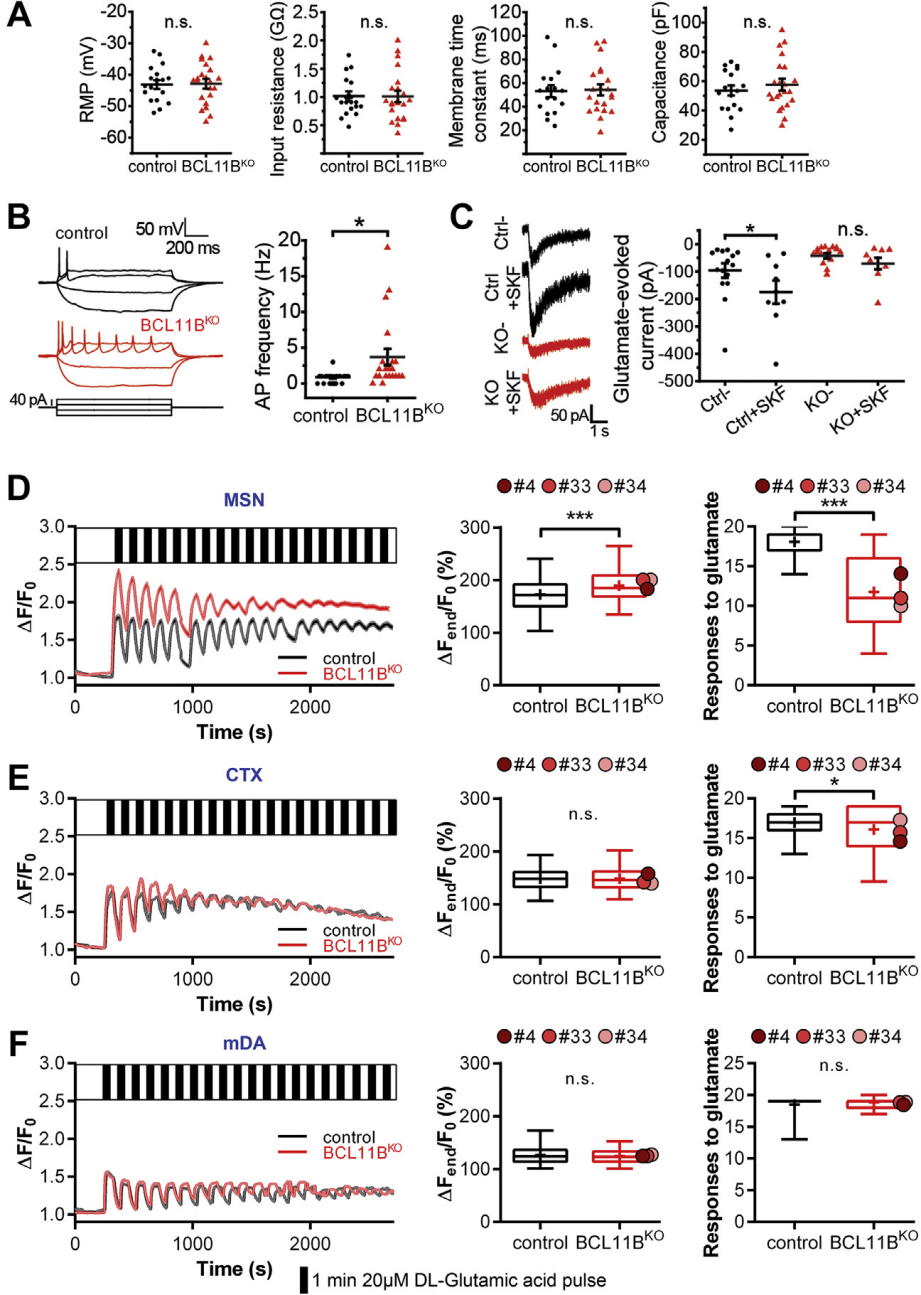
BCL11B^KO^ MSNs exhibit no dopaminergic modulation of excitatory signaling and present with impaired glutamate-evoked Ca^2+^ signals. **(A)** All basic membrane properties, including RMP, input resistance, membrane time constant, and capacitance, were measured and found to not differ significantly between the genotypes. **(B)** Representative traces of action potentials evoked by steps of current injection in Ctrl (black) and BCL11B^KO^ (red) MSNs (left). Increased firing frequency in response to current injection is observed in BCL11B^KO^ MSNs (right) (Mann-Whitney *U* test: *U* = 90.5, ∗*p* = .014). **(C)** Representative traces and quantification of current responses to DL-glutamic acid (200 μM, 30 ms) in the absence or presence of SKF-81297 (SKF) (10 μM) in Ctrl and BCL11B^KO^ MSNs. Only Ctrl MSNs show a significant enhancement of glutamatergic transmission induced by activation of dopaminergic signaling (two-way analysis of variance with post hoc Bonferroni test: genotype: *F*_1,40_ = 8.860, *p* = .005; treatment: *F*_1,40_ = 4.093, *p* = .049; Ctrl vs. KO: *p* = .123, Ctrl vs. Ctrl + SKF: ∗*p* = .038, KO vs. KO + SKF: *p* = .463, Ctrl + SKF vs. KO + SKF: ∗*p* = .014). **(D–F)** Glutamate-evoked intracellular Ca^2+^ signals recorded in response to repetitive stimulation (20 μM, 1 minute, 19 pulses, black bars) in control and BCL11B^KO^ MSNs, CTX neurons, and mDA neurons (left column). **(D)** Glutamate-stimulated BCL11B^KO^ MSNs accumulate higher intracellular Ca^2+^ levels over time and respond to fewer glutamate pulses compared with Ctrl cells (Mann-Whitney *U* test: MSN Δ*F*_*end*_*/F*_*0*_: *U* = 621,047, ∗∗∗*p* = 1.8 × 10^−25^; responses: *U* = 117,472, ∗∗∗*p* = 2.8 × 10^−153^). **(E, F)** Only a small deficit is present in the number of glutamate-evoked responses in BCL11B^KO^ CTX neurons, while no differences can be seen in mDA neurons (Mann-Whitney *U* test: CTX responses: *U* = 287,590, ∗*p* = .043). All line graphs and dot plots depict mean ± SEM for each genotype. Box-and-whisker plots depict data for each genotype (center line, median; +, mean; box limits, upper and lower quartiles; whiskers, 2.5 and 97.5 percentiles). Means for individual clones are indicated by red-shaded circles next to BCL11B^KO^ data. See also Figure S4. AP, action potential; Ctrl, control; CTX, cortical glutamatergic; KO, knockout; mDA, midbrain dopamine; MSNs, medium spiny neurons; n.s., not significant; RMP, resting membrane potential.

Glutamatergic transmission in MSNs is known to be enhanced by dopamine acting on postsynaptic DRD1 (38). Considering abnormal dopaminergic and glutamatergic synaptic signaling in BCL11B-deficient MSNs suggested in a prior study (11), we next inspected physiological implications of this by performing patch-clamp electrophysiology in MSN cultures at 40 DIV. While both control and BCL11B^KO^ neurons displayed similar basic membrane properties, firing frequency was markedly higher in BCL11B^KO^ MSNs (Figure 3A, B). Glutamate-evoked currents were not significantly different between BCL11B^KO^ and control cells at baseline; however, only control MSNs showed a significant increase in the current amplitude following treatment with DRD1/DRD5 agonist (Figure 3C, Figure S4A).

Intracellular Ca^2+^ is delicately balanced and plays an indisputable role in determining neuronal excitability. Therefore, we next tested the effect of BCL11B loss on neuron response to excitatory stimulation by measuring ΔCa^2+^ evoked by glutamate pulses in MSNs, cortical neurons, and mDA neurons (Figure 3D–F). BCL11B^KO^ MSNs initially exhibited larger glutamate-evoked ΔCa^2+^ but desensitized faster than control MSNs, a sign of excitotoxicity (Figure 3D). Indeed, significantly greater increases in intracellular Ca^2+^ levels over time together with fewer glutamate-evoked responses were observed in BCL11B^KO^ MSNs compared with control cells. This phenotype was mostly specific to MSNs because only a small deficit was detected in the number of glutamate-evoked responses in BCL11B^KO^ cortical neurons (Figure 3E), while no differences were seen in mDA neurons due to loss of BCL11B (Figure 3F).

A key measurement of neuronal functional maturity is the development of complex morphological features such as dendritic branching. Analysis of neuron morphology revealed no striking differences between control and BCL11B^KO^ MSNs (Figure S4B–E). In conclusion, although BCL11B-deficient MSNs were largely comparable to control cells in membrane properties and morphology, they presented with significantly impaired responsiveness to physiological stimuli, including glutamate-evoked Ca^2+^ signaling and DRD1-mediated modulation of glutamate-evoked currents, a feature characteristic of MSNs in vivo (38).

### cAMP-PKA-Calcium Signaling Axis Is Driven by BCL11B-Dependent Transcription Programs

**Figure 4:**
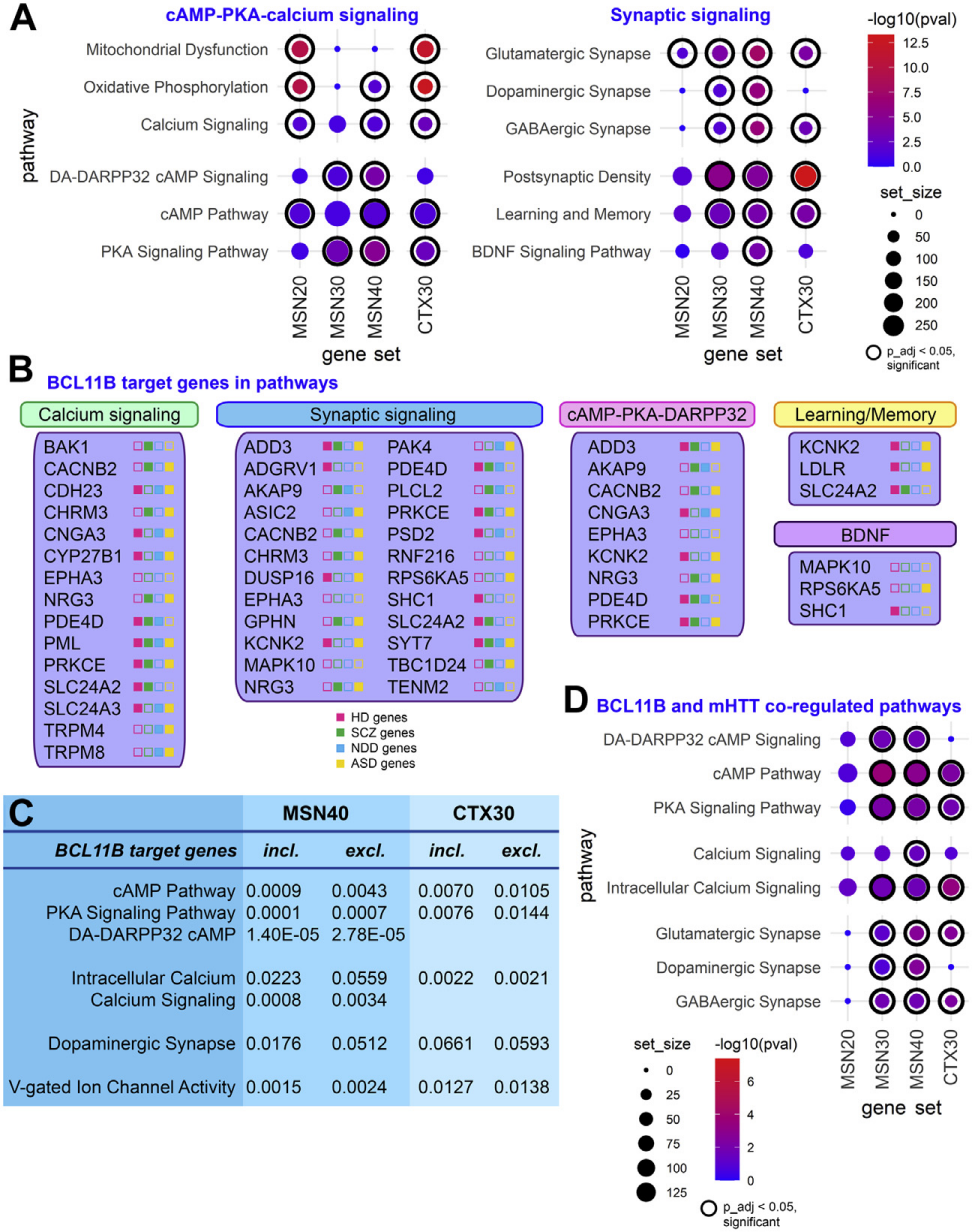
BCL11B-regulated transcription programs in MSNs and CTX neurons overlap with mHTT–coregulated pathways. **(A)** Kyoto Encyclopedia of Genes and Genomes and Ingenuity Pathway Analysis of differentially expressed genes at false discovery rate *p*_*adj*_ < .01 shows a significant enrichment of genes regulating cAMP-PKA-calcium signaling axis and synaptic signaling in both neuron types as well as DA-DARPP32 signaling specifically in MSNs (full gene set lists are presented in Table S2). **(B)** BCL11B target genes within shortlisted core biological processes affected by the loss of BCL11B in MSN and CTX neurons. Next to gene name, color icons indicate significant association (differential expression or identified risk variant) with HD (pink), SCZ (green), NDD (blue), and ASD (yellow). **(C)** Exclusion of BCL11B target genes from differentially expressed gene list results in several pathways in MSNs becoming either drastically less significant or not significant at all, including cAMP-PKA-calcium signaling axis pathways and dopamine synapse signaling, while having almost no effect on signaling pathways in CTX neurons. **(D)** BCL11B-regulated signaling abnormalities in MSNs and CTX neurons are shared by HD neurons (full gene set lists are presented in Table S4). In pathway graphs **(A, D)**, dot size corresponds to gene set size, dot color corresponds to −log_10_(*pval*), where *p*_adj < .05 dot is framed inside a black circle. See also Figures S5 and S6 and Tables S1–S4. ASD, autism spectrum disorder; BDNF, brain-derived neurotrophic factor; CTX, cortical glutamatergic; DA, dopamine; excl., exclusion; GABA, gamma-aminobutyric acid; HD, Huntington’s disease; incl., inclusion; mHTT, mutant huntingtin; MSNs, medium spiny neurons; NDD, neurodevelopmental disorder; *p*_adj, *p* adjusted; PKA, protein kinase A; SCZ, schizophrenia.

To investigate molecular mechanisms and pathways leading to pathological changes in BCL11B^KO^ MSNs and cortical neurons, we performed whole-transcriptome RNA sequencing analysis at different stages of differentiation (Table S1). To elucidate specific biological processes regulated by BCL11B, we performed Ingenuity Pathway Analysis and Kyoto Encyclopedia of Genes and Genomes pathway analysis of protein-coding differentially expressed genes (DEGs) (false discovery rate *p*_*adj*_ < .01). Genes regulating calcium signaling, mitochondrial function, and oxidative phosphorylation were found to be significantly altered in both neuronal types (Figure 4A, Table S2). Moreover, DA-DARPP32 feedback in cAMP signaling deficits was specific to the MSN population, while PKA and cAMP pathway dysregulation was stronger in MSNs but also present in cortical neurons. Furthermore, synaptic genes regulating dopamine, glutamate, and GABA neurotransmitters were significantly altered in MSNs from as early as 20 and 30 DIV. In contrast, only glutamatergic and GABAergic synapse signaling was affected in cortical neurons. In line with previously identified roles for BCL11B in the brain (3,4), we also observed significant dysregulation of BDNF (brain-derived neurotrophic factor) signaling specifically in MSNs, as well as abnormal learning and memory signaling in both neuronal types.

To further elucidate the role of BCL11B in the identified pathogenic pathways, we asked whether the enriched pathways are driven by BCL11B target genes (Figure 4B, Figure S5). Once BCL11B target genes were excluded from the DEG list, several pathways in MSNs became either drastically less significant or not significant at all, including cAMP-PKA-calcium signaling axis pathways and dopamine synapse signaling (Figure 4C, Table S2). While BCL11B target genes appeared to play a weak role in cAMP-PKA signaling in cortical neurons, their exclusion did not affect calcium or dopamine synapse signaling in these cells. Transcriptomic data corroborates cellular pathologies identified in knockout neurons, suggesting that BCL11B plays a role in mitochondrial and intracellular Ca^2+^ signaling in both neuronal types, while its MSN-specific role converges on a few select pathways primarily regulating the cAMP-PKA-calcium signaling axis and downstream glutamate/DA-DARPP32 neurotransmission.

### BCL11B-Regulated Signaling Abnormalities Are Shared by HD Neurons

BCL11B hypofunction has previously been demonstrated to play a role in MSN degeneration in HD by regulating mitochondrial signaling and protein phosphorylation (8,11). We therefore investigated potential overlap between BCL11B- and mHTT-mediated transcriptomic changes by comparing our DEGs in both neuronal types with four publicly available datasets from human and mouse HD models (Figure S6) (7,10,39,40). Gene set enrichment analysis indeed revealed that all MSN and cortical DEGs were significantly enriched for mHTT-regulated genes (Table S3). Further investigation of concordantly dysregulated genes between BCL11B^KO^ and HD models confirmed deficits in DA-DARPP32 signaling, cAMP-PKA-calcium signaling axis, synaptic signaling, oxidative phosphorylation, and mitochondrial function predominantly in MSNs and to a lesser extent in cortical neurons (Figure 4D, Table S4). Multiple BCL11B target genes in these pathways have been previously shown to be affected in HD (Figure 4B). These results provide further support for a regulatory role for BCL11B in the cAMP-PKA-calcium signaling axis and downstream DA-DARPP32 neurotransmission events, processes that are particularly vulnerable to mHTT.

### A Role for cAMP-PKA-Calcium Signaling Axis in the Neuropathology of Psychiatric Disorders

A direct role for MSNs in psychiatric disease pathogenesis has been suggested by recent gene enrichment studies (19, 20, 21). Moreover, independent studies have fine-mapped and prioritized the *BCL11B* gene among a few others as a candidate causal SCZ risk gene (17,20,41). Considering these reports, we wonder whether the BCL11B-dependent neuronal phenotypes described in this study may contribute to pathogenesis in psychiatric conditions. To gain support of this hypothesis, we investigated BCL11B-regulated genes in MSNs and cortical neurons, with particular interest in the signaling pathways responsible for the observed convergent and MSN-specific deficits, and their enrichment for risk variants in SCZ, neurodevelopmental disorder (NDD), and autism spectrum disorder (ASD). Neurologic disease gene sets were collated by integrating findings from transcriptomics, genome-wide association study datasets, and other functional genomics studies of SCZ (13,14,20,21,42, 43, 44, 45, 46, 47, 48, 49, 50, 51, 52, 53, 54), NDD (51,53,55), and ASD (46,53,56) (Figure S6, Table S5). In line with neurodevelopmental components reported in SCZ and ASD, an overlap of about one third of genes was detected between these two conditions and the NDD gene set.

**Figure 5:**
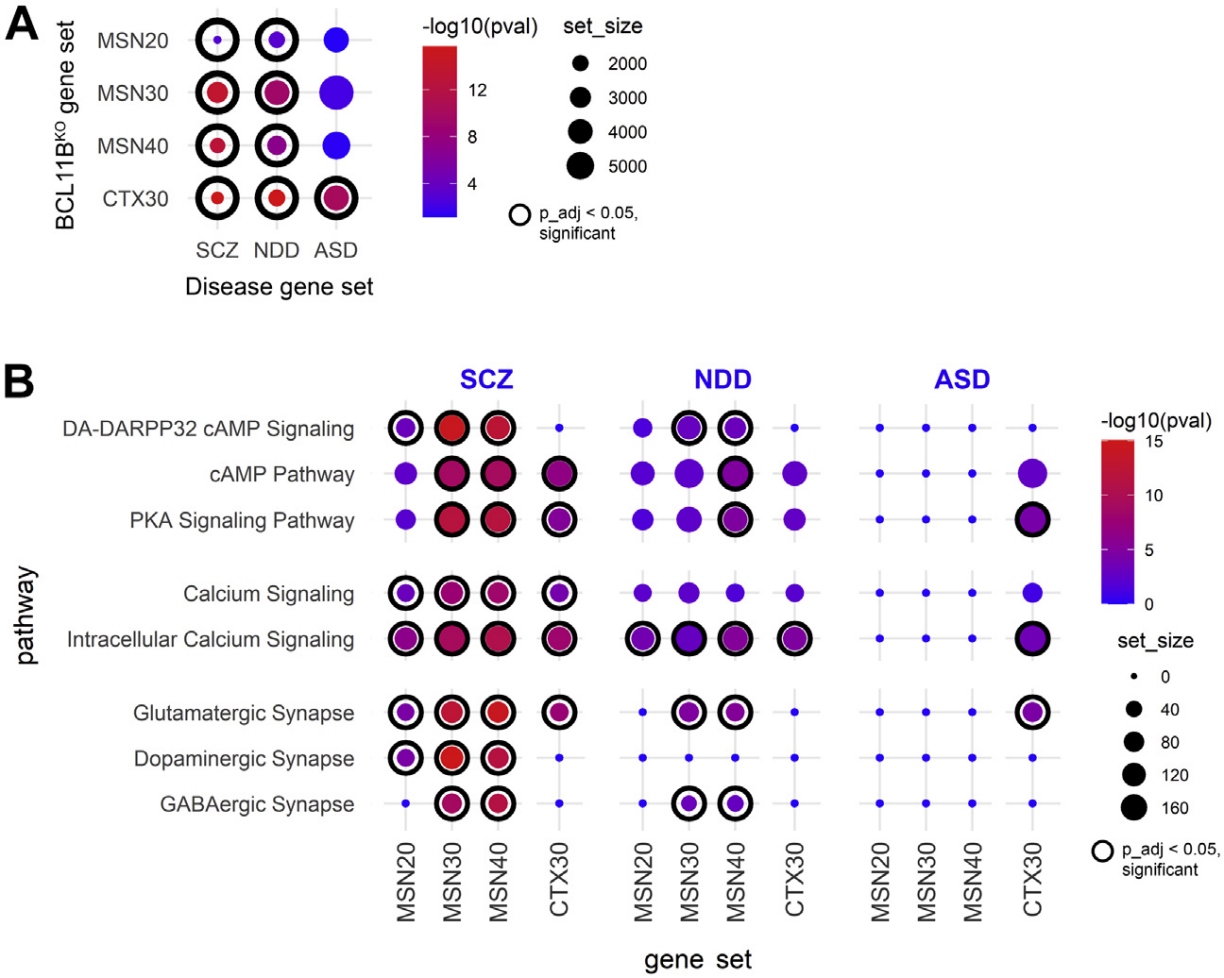
A role for BCL11B-dependent DA-DARPP32 and cAMP-PKA-calcium signaling axis in the pathogenesis of psychiatric disorders. **(A)** BCL11B-regulated genes in MSNs are significantly enriched for SCZ and NDD risk genes but not ASD risk genes at all stages of differentiation, while CTX30 differentially expressed genes were enriched for all disease risk genes. *p* values were calculated from Fisher’s exact test followed by Bonferroni correction for multiple comparisons. **(B)** BCL11B-dependent signaling pathways in MSNs are predominantly enriched for SCZ but not NDD risk variants, while pathways in CTX neurons were enriched for both SCZ and ASD risk genes (full gene set lists are presented in Table S7). In graphs, dot size corresponds to gene set size, dot color corresponds to −log_10_(*pval*), where *p*_adj < .05 dot is framed inside a black circle. See also Figures S5 and S6 and Tables S5–S7. ASD, autism spectrum disorder; CTX, cortical glutamatergic; DA, dopamine; GABA, gamma-aminobutyric acid; KO, knockout; MSNs, medium spiny neurons; NDD; neurodevelopmental disorder; *p*_adj, *p* adjusted; PKA, protein kinase A; SCZ, schizophrenia.

Gene set enrichment analysis revealed that BCL11B^KO^ MSN DEGs were enriched for SCZ and NDD risk genes but not ASD risk genes at all stages of differentiation, while CTX30 DEGs were enriched for all disease risk genes (Figure 5A, Table S6). In MSN cultures, BCL11B-dependent enrichment of DA-DARPP32 and cAMP-PKA-calcium signaling functional terms was much stronger and appeared earlier for SCZ than NDD risk genes (Figure 5B, Table S7). Furthermore, SCZ risk variants were enriched in synaptic signaling affecting all three neurotransmitters, while NDD risk genes were present only in glutamatergic and GABAergic synaptic pathways. In cortical cultures, dysregulated pathways were more enriched for SCZ and ASD than NDD risk variants. Most BCL11B target genes in these pathways have been previously implicated in at least one of these three neurologic disorders (Figure 4B). Together, these findings suggest that synaptic signaling and cAMP-PKA-calcium signaling axis dysregulation may contribute to SCZ neuropathology in the striatum. In contrast, in cortical neurons, some of these BCL11B-dependent phenotypes, although affected less severely, may contribute to both SCZ and ASD pathophysiology.

## Discussion

**Figure 6:**
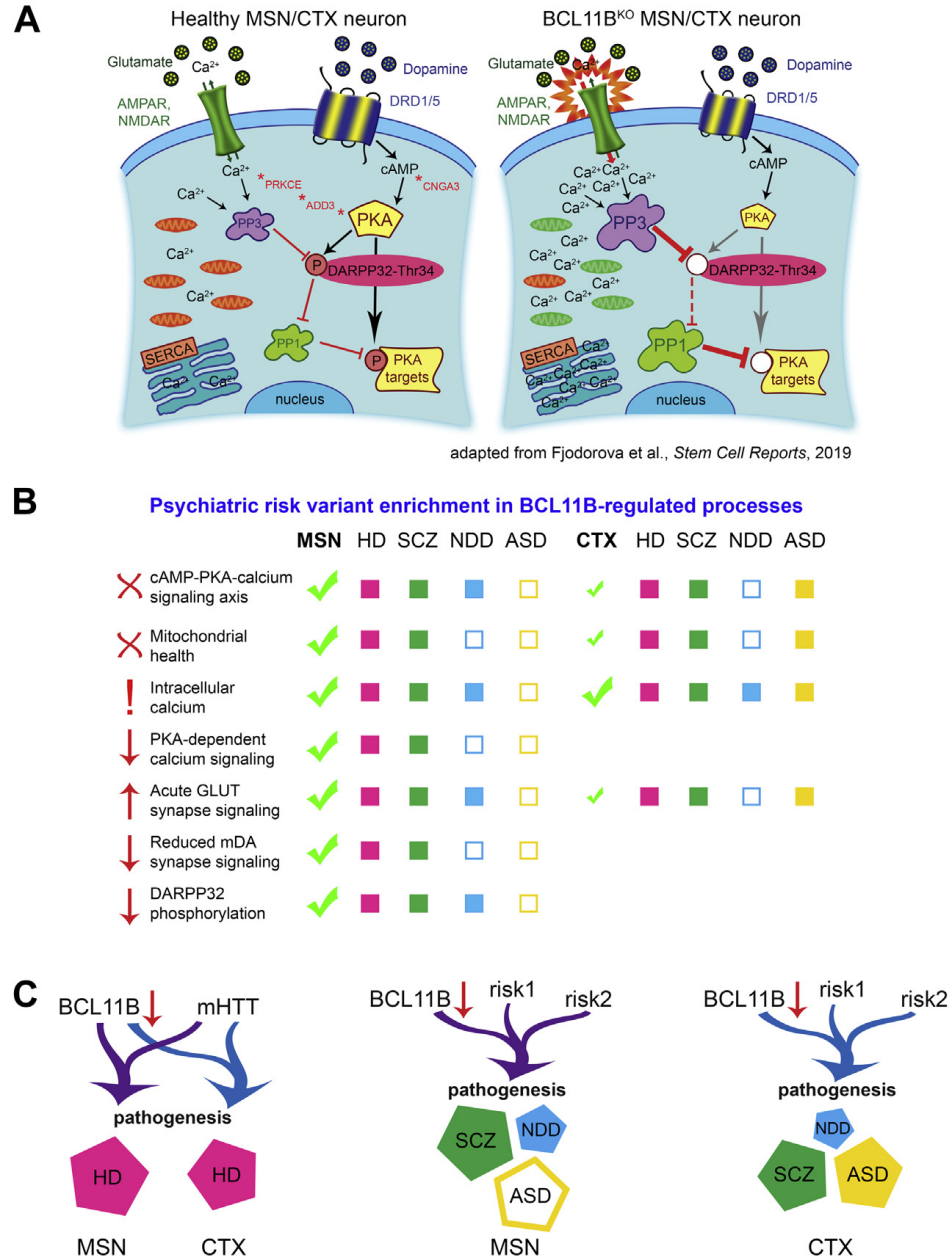
BCL11B-regulated molecular events in MSNs and CTX neurons and their likely contribution to psychiatric disorder pathogenesis. **(A)** Schematic of core biological processes affected by the loss of BCL11B summarizes phenotypes identified in current and previous studies, including depolarized mitochondria (green), abnormal calcium signaling, acute glutamatergic neurotransmission, disbalance in phosphatase levels, reduced cAMP-PKA signaling, and downstream dephosphorylation of DARPP32 and other targets [adapted from (11)]. **(B)** These core molecular changes are most prominent in MSNs compared with CTX neurons and show preferential enrichment for HD and SCZ genes, suggesting that they are likely to contribute to the pathogenesis in these disorders. In contrast, in CTX neurons, a fraction of these BCL11B-dependent phenotypes, although affected less severely, may also contribute to ASD pathogenesis. **(C)** In summary, we propose that MSNs play a distinct role in psychiatric disease development compared with CTX neurons, where BCL11B hypofunction acts in tandem with mHTT in HD and other risk variants in psychiatric disorders to contribute to disease pathogenesis. AMPAR, AMPA receptor; ASD, autism spectrum disorder; CTX, cortical glutamatergic; HD, Huntington’s disease; KO, knockout; mDA, midbrain dopaminergic; mHTT, mutant huntingtin; MSNs, medium spiny neurons; NDD, neurodevelopmental disorder; NMDAR, NMDA receptor; PKA, protein kinase A; SCZ, schizophrenia.

In this study, we provide evidence supporting a role for corticostriatal transcription factor BCL11B predominantly in HD and SCZ than in NDD and ASD neuropathology. We further strengthen the hypothesis that MSN dysfunction may contribute separately from cortical neuron pathology to psychiatric disease development. Such neuron subtype and multiple disorder cross-examination allows the opportunity to study gene expression patterns and determine convergent versus distinct BCL11B-regulated mechanisms contributing to pathogenesis of different diseases. Indeed, our gene enrichment analysis and functional assays reveals a limited number of core biological processes affected by the loss of BCL11B that are highly specific to the MSN population, such as the cAMP-PKA-calcium signaling axis, DA-DARPP32 signaling, glutamate-evoked calcium signaling, and mitochondrial health (Figure 6). Intracellular Ca^2+^ signaling deficit is the only phenotype common between MSNs and cortical neurons, while other calcium and mitochondrial signaling deficits are only mildly present in cortical neurons. Transcriptomic analysis suggests that identified BCL11B-dependent biological processes are mainly concordant between HD and SCZ in MSNs, while cortical neuron pathways were also enriched for ASD risk variants. Furthermore, our study predicts involvement of BCL11B target genes in regulating these pathways, with many genes identified either as risk factors for or differentially expressed in psychiatric disorders.

BCL11B target genes appear to drive cAMP-PKA-calcium signaling axis and dopamine receptor signaling pathways predominantly in MSNs. The most notable genes among them are *ADD3*, *CACNB2*, *CNGA3*, *KCNK2*, and *SYT7*, with significant downregulation of *ADD3* and *CNGA3* already confirmed in independent BCL11B^KO^ samples in our previous study (11). These five genes play a role in activation of cAMP-PKA-calcium signaling, synaptic signaling, and excitation of neurons (57, 58, 59, 60, 61). Furthermore, they are either dysregulated in or considered to be risk factors for multiple neurologic diseases, including HD, SCZ, NDD, ASD, and bipolar disorder (7,10,13,39,47,55,62, 63, 64, 65, 66). Together, this evidence provides potential mechanistic insight into how BCL11B hypofunction may contribute to the disturbed cAMP-PKA-calcium and synaptic signaling in MSNs in HD and SCZ.

Transgenic expression of *Bcl11b* in ST*Hdh*^*Q111*^ HD cells (that exhibit reduced levels of BCL11B) partially rescued mHTT-induced defects in mitochondrial metabolic activity (8). This suggests that restoring or increasing BCL11B levels can reverse at least some of the mHTT-driven impairments. Mitochondrial dysfunction and elevated intracellular Ca^2+^ levels are highly interdependent phenotypes in neurodegenerative diseases (32). Similar to HD cells, BCL11B-deficient neurons present with mitochondrial deficits, vulnerability to oxidative stress, and abnormal regulation of intracellular Ca^2+^ levels. While our results cannot definitively discriminate between increased Ca^2+^ influx and other mechanisms, such as impaired Ca^2+^ buffering, our data show that regulation of Ca^2+^ levels is impaired by the loss of BCL11B, and this could lead to intracellular Ca^2+^ overload, a proposed critical step in neurodegeneration (67). Moreover, the same deficits in mitochondrial membrane potential as reported for BCL11B^KO^ MSNs here have been previously described in cortical neurons derived from patients with SCZ (68). Although no deficits in either electrophysiological or spontaneous ΔCa^2+^ activity were found in cortical neurons in this study (68), reduced electrical activity in hippocampal CA3 neurons derived from patients with SCZ has been reported elsewhere (69,70), while no studies in patient MSNs have been conducted yet. We demonstrate that BCL11B-dependent oxidative phosphorylation as well as calcium signaling in MSNs are enriched for SCZ risk genes. This suggests that BCL11B-associated phenotypes may contribute to neuropathology in SCZ and that there might be yet undiscovered MSN dysfunction in this psychiatric disease development.

A connection between disturbed calcium signaling, excitatory stimulation, and MSN apoptosis has been established in HD models (34,71, 72, 73, 74, 75). We demonstrate that glutamate induces elevated Ca^2+^ responses in BCL11B^KO^ MSNs, a feature of excitotoxicity, which suggests that BCL11B hypofunction might be the cause of aberrant calcium signaling and MSN apoptosis observed in HD. Although BCL11B knockout appears to enhance intrinsic excitability of MSNs, it simultaneously impairs dopaminergic modulation of glutamate-mediated excitation. Together, these results provide strong evidence of a central role for BCL11B in regulating calcium homeostasis in MSNs and, to some extent, in cortical neurons, the disruption of which in BCL11B-deficient and HD MSNs induces Ca^2+^ overload, leading to excitotoxicity and pathological responses to physiological stimuli. These BCL11B-associated glutamatergic and dopaminergic signaling phenotypes may also be present in MSNs in SCZ.

Furthermore, PKA signaling was previously implicated in modulating intracellular Ca^2+^ levels and Ca^2+^ oscillations (35,76). In agreement with these findings, we demonstrate that deficits in spontaneous activity–evoked Ca^2+^ oscillations in BCL11B-deficient MSNs can be rescued in a PKA-dependent manner. Thus, we propose that BCL11B deficiency–driven disruption of calcium homeostasis in MSNs, together with dysregulated levels of protein phosphatases/kinases (11), result from reduced PKA signaling and lead to a marked decrease in phosphorylation of its targets. In addition, increased intracellular Ca^2+^ levels would induce overactivation of PP3-dependent DARPP32-Thr34 dephosphorylation (36). Identified disruption of PKA-regulated intracellular Ca^2+^ signaling and phosphorylation of DARPP32 in MSNs has significant implications for many psychiatric and neurodegenerative diseases (11,77,78). These processes regulate transcriptional and behavioral responses of MSNs to pharmacological stimuli, including antidepressants, neuroleptics, and drugs of abuse (79). Reduced levels of full-length DARPP32 and increased levels of DARPP32 isoforms lacking the crucial residue Thr34 were reported in patients with SCZ (80,81), which suggests disruption of DARPP32-Thr34 phosphorylation in SCZ MSNs. Moreover, abnormal postsynaptic PKA activity due to accelerated maturation of corticostriatal circuits was demonstrated to cause behavioral abnormalities in a *Shank3B*^*−/−*^ mouse model of ASD (82). Indeed, we demonstrate that BCL11B-dependent cAMP-PKA-calcium signaling and DA-DARPP32 signaling pathways in MSNs are enriched for psychiatric disorder risk variants, pointing to a potential role for BCL11B-associated phenotypes in neuropathology in these disorders.

In conclusion, we identify BCL11B-regulated molecular mechanisms in striatal and cortical neurons and further strengthen the hypothesis that MSN dysfunction may contribute separately from cortical neuron pathology to psychiatric disease development. We provide evidence that genetic susceptibility loci within BCL11B-regulated pathways may modulate a limited number of core disease-related biological processes in MSNs, including cAMP-PKA-calcium signaling, DA-DARPP32 signaling, and glutamate neurotransmission. We propose that BCL11B-associated phenotypes may contribute to neuropathology most significantly in HD and SCZ and identify modulation of PKA-dependent Ca^2+^ signals and protein phosphorylation as potential new therapeutic targets in the striatum.

## Supplementary Material

Table S7

Key Resources Table

## References

[1] Arlotta P., Molyneaux B.J., Jabaudon D., Yoshida Y., Macklis J.D. (2008). Ctip2 controls the differentiation of medium spiny neurons and the establishment of the cellular architecture of the striatum. J Neurosci.

[2] Victor M.B., Richner M., Hermanstyne T.O., Ransdell J.L., Sobieski C., Deng P.Y. (2014). Generation of human striatal neurons by microRNA-dependent direct conversion of fibroblasts. Neuron.

[3] Tang B., Di Lena P., Schaffer L., Head S.R., Baldi P., Thomas E.A. (2011). Genome-wide identification of Bcl11b gene targets reveals role in brain-derived neurotrophic factor signaling. PLoS One.

[4] Simon R., Baumann L., Fischer J., Seigfried F.A., De Bruyckere E., Liu P. (2016). Structure-function integrity of the adult hippocampus depends on the transcription factor Bcl11b/Ctip2. Genes Brain Behav.

[5] Srinivasan K., Leone D.P., Bateson R.K., Dobreva G., Kohwi Y., Kohwi-Shigematsu T. (2012). A network of genetic repression and derepression specifies projection fates in the developing neocortex. Proc Natl Acad Sci U S A.

[6] Arlotta P., Molyneaux B.J., Chen J., Inoue J., Kominami R., Macklis J.D. (2005). Neuronal subtype-specific genes that control corticospinal motor neuron development in vivo. Neuron.

[7] Ring K.L., An M.C., Zhang N., O’Brien R.N., Ramos E.M., Gao F. (2015). Genomic analysis reveals disruption of striatal neuronal development and therapeutic targets in human Huntington’s disease neural stem cells. Stem Cell Reports.

[8] Desplats P.A., Lambert J.R., Thomas E.A. (2008). Functional roles for the striatal-enriched transcription factor, Bcl11b, in the control of striatal gene expression and transcriptional dysregulation in Huntington’s disease. Neurobiol Dis.

[9] Ahmed I., Sbodio J.I., Harraz M.M., Tyagi R., Grima J.C., Albacarys L.K. (2015). Huntington’s disease: Neural dysfunction linked to inositol polyphosphate multikinase. Proc Natl Acad Sci U S A.

[10] Langfelder P., Cantle J.P., Chatzopoulou D., Wang N., Gao F.Y., Al-Ramahi I. (2016). Integrated genomics and proteomics define huntingtin CAG length-dependent networks in mice. Nat Neurosci.

[11] Fjodorova M., Louessard M., Li Z., De La Fuente D.C., Dyke E., Brooks S.P. (2019). CTIP2-regulated reduction in PKA-dependent DARPP32 phosphorylation in human medium spiny neurons: Implications for Huntington disease. Stem Cell Reports.

[12] Lessel D., Gehbauer C., Bramswig N.C., Schluth-Bolard C., Venkataramanappa S., van Gassen K.L.I. (2018). BCL11B mutations in patients affected by a neurodevelopmental disorder with reduced type 2 innate lymphoid cells. Brain.

[13] Schizophrenia Working Group of the Psychiatric Genomics Consortium (2014). Biological insights from 108 schizophrenia-associated genetic loci. Nature.

[14] Pardiñas A.F., Holmans P., Pocklington A.J., Escott-Price V., Ripke S., Carrera N. (2018). Common schizophrenia alleles are enriched in mutation-intolerant genes and in regions under strong background selection [published correction appears in Nat Genet 2019; 51:1193]. Nat Genet.

[15] Wu Y., Cao H., Baranova A., Huang H., Li S., Cai L. (2020). Multi-trait analysis for genome-wide association study of five psychiatric disorders [published correction appears in Transl Psychiatry 2020; 10:234]. Transl Psychiatry.

[16] Yao X., Glessner J.T., Li J., Qi X., Hou X., Zhu C. (2021). Integrative analysis of genome-wide association studies identifies novel loci associated with neuropsychiatric disorders. Transl Psychiatry.

[17] Trubetskoy V., Pardiñas A.F., Qi T., Panagiotaropoulou G., Awasthi S., Bigdeli T.B. (2022). Mapping genomic loci implicates genes and synaptic biology in schizophrenia. Nature.

[18] Jahangir M., Zhou J.S., Lang B., Wang X.P. (2021). GABAergic system dysfunction and challenges in schizophrenia research [published correction appears in Front Cell Dev Biol 2021; 9:742519]. Front Cell Dev Biol.

[19] Skene N.G., Bryois J., Bakken T.E., Breen G., Crowley J.J., Gaspar H.A. (2018). Genetic identification of brain cell types underlying schizophrenia. Nat Genet.

[20] Gamazon E.R., Zwinderman A.H., Cox N.J., Denys D., Derks E.M. (2019). Multi-tissue transcriptome analyses identify genetic mechanisms underlying neuropsychiatric traits. Nat Genet.

[21] Huckins L.M., Dobbyn A., Ruderfer D.M., Hoffman G., Wang W., Pardiñas A.F. (2019). Gene expression imputation across multiple brain regions provides insights into schizophrenia risk [published correction appears in Nat Genet 2019; 51:1068]. Nat Genet.

[22] Jaeger I., Arber C., Risner-Janiczek J.R., Kuechler J., Pritzsche D., Chen I.C. (2011). Temporally controlled modulation of FGF/ERK signaling directs midbrain dopaminergic neural progenitor fate in mouse and human pluripotent stem cells. Development.

[23] Arber C., Precious S.V., Cambray S., Risner-Janiczek J.R., Kelly C., Noakes Z. (2015). Activin A directs striatal projection neuron differentiation of human pluripotent stem cells. Development.

[24] Fjodorova M., Noakes Z., Li M. (2020). A role for TGFβ signalling in medium spiny neuron differentiation of human pluripotent stem cells. Neuronal Signal.

[25] Patel T.P., Man K., Firestein B.L., Meaney D.F. (2015). Automated quantification of neuronal networks and single-cell calcium dynamics using calcium imaging. J Neurosci Methods.

[26] Telezhkin V., Schnell C., Yarova P., Yung S., Cope E., Hughes A. (2016). Forced cell cycle exit and modulation of GABA(A), CREB, and GSK3 beta signaling promote functional maturation of induced pluripotent stem cell-derived neurons. Am J Physiol Cell Physiol.

[27] Love M.I., Huber W., Anders S. (2014). Moderated estimation of fold change and dispersion for RNA-seq data with DESeq2. Genome Biol.

[28] Yu G., Wang L.G., Han Y., He Q.Y. (2012). clusterProfiler: An R package for comparing biological themes among gene clusters. OMICS.

[29] Manoharan S., Guillemin G.J., Abiramasundari R.S., Essa M.M., Akbar M., Akbar M.D. (2016). The role of reactive oxygen species in the pathogenesis of Alzheimer’s disease, Parkinson’s disease, and Huntington’s disease: A mini review. Oxid Med Cell Longev.

[30] Valdez L.B., Zaobornyj T., Boveris A. (2006). Mitochondrial metabolic states and membrane potential modulate mtNOS activity. Biochim Biophys Acta.

[31] Wei T., Chen C., Hou J., Xin W., Mori A. (2000). Nitric oxide induces oxidative stress and apoptosis in neuronal cells. Biochim Biophys Acta.

[32] Wang J.Q., Chen Q., Wang X., Wang Q.C., Wang Y., Cheng H.P. (2013). Dysregulation of mitochondrial calcium signaling and superoxide flashes cause mitochondrial genomic DNA damage in Huntington disease. J Biol Chem.

[33] Wu J., Shih H.P., Vigont V., Hrdlicka L., Diggins L., Singh C. (2011). Neuronal store-operated calcium entry pathway as a novel therapeutic target for Huntington’s disease treatment. Chem Biol.

[34] Nekrasov E.D., Vigont V.A., Klyushnikov S.A., Lebedeva O.S., Vassina E.M., Bogomazova A.N. (2016). Manifestation of Huntington’s disease pathology in human induced pluripotent stem cell-derived neurons. Mol Neurodegener.

[35] Tang T.S., Bezprozvanny I. (2004). Dopamine receptor-mediated Ca(2+) signaling in striatal medium spiny neurons. J Biol Chem.

[36] Nishi A., Snyder G.L., Nairn A.C., Greengard P. (1999). Role of calcineurin and protein phosphatase-2A in the regulation of DARPP-32 dephosphorylation in neostriatal neurons. J Neurochem.

[37] Bibb J.A., Snyder G.L., Nishi A., Yan Z., Meijer L., Fienberg A.A. (1999). Phosphorylation of DARPP-32 by Cdk5 modulates dopamine signalling in neurons. Nature.

[38] Flores-Hernández J., Cepeda C., Hernández-Echeagaray E., Calvert C.R., Jokel E.S., Fienberg A.A. (2002). Dopamine enhancement of NMDA currents in dissociated medium-sized striatal neurons: Role of D1 receptors and DARPP-32. J Neurophysiol.

[39] Hodges A., Strand A.D., Aragaki A.K., Kuhn A., Sengstag T., Hughes G. (2006). Regional and cellular gene expression changes in human Huntington’s disease brain. Hum Mol Genet.

[40] HD iPSC Consortium (2017). Developmental alterations in Huntington’s disease neural cells and pharmacological rescue in cells and mice. Nat Neurosci.

[41] Whitton L., Cosgrove D., Clarkson C., Harold D., Kendall K., Richards A. (2016). Cognitive analysis of schizophrenia risk genes that function as epigenetic regulators of gene expression. Am J Med Genet B Neuropsychiatr Genet.

[42] Schizophrenia Psychiatric Genome-Wide Association Study (GWAS) Consortium (2011). Genome-wide association study identifies five new schizophrenia loci. Nat Genet.

[43] Ayalew M., Le-Niculescu H., Levey D.F., Jain N., Changala B., Patel S.D. (2012). Convergent functional genomics of schizophrenia: From comprehensive understanding to genetic risk prediction. Mol Psychiatry.

[44] Kirov G., Pocklington A.J., Holmans P., Ivanov D., Ikeda M., Ruderfer D. (2012). De novo CNV analysis implicates specific abnormalities of postsynaptic signalling complexes in the pathogenesis of schizophrenia. Mol Psychiatry.

[45] Ripke S., O’Dushlaine C., Chambert K., Moran J.L., Kähler A.K., Akterin S. (2013). Genome-wide association analysis identifies 13 new risk loci for schizophrenia. Nat Genet.

[46] Fromer M., Pocklington A.J., Kavanagh D.H., Williams H.J., Dwyer S., Gormley P. (2014). De novo mutations in schizophrenia implicate synaptic networks. Nature.

[47] Purcell S.M., Moran J.L., Fromer M., Ruderfer D., Solovieff N., Roussos P. (2014). A polygenic burden of rare disruptive mutations in schizophrenia. Nature.

[48] Rees E., Carrera N., Morgan J., Hambridge K., Escott-Price V., Pocklington A.J. (2019). Targeted sequencing of 10,198 samples confirms abnormalities in neuronal activity and implicates voltage-gated sodium channels in schizophrenia pathogenesis. Biol Psychiatry.

[49] Liu C., Kanazawa T., Tian Y., Mohamed Saini S., Mancuso S., Mostaid M.S. (2019). The schizophrenia genetics KnowledgeBase: A comprehensive update of findings from candidate gene studies. Transl Psychiatry.

[50] Schijven D., Kofink D., Tragante V., Verkerke M., Pulit S.L., Kahn R.S. (2018). Comprehensive pathway analyses of schizophrenia risk loci point to dysfunctional postsynaptic signaling. Schizophr Res.

[51] Rees E., Han J., Morgan J., Carrera N., Escott-Price V., Pocklington A.J. (2020). De novo mutations identified by exome sequencing implicate rare missense variants in SLC6A1 in schizophrenia. Nat Neurosci.

[52] Lam M., Chen C.Y., Li Z., Martin A.R., Bryois J., Ma X. (2019). Comparative genetic architectures of schizophrenia in East Asian and European populations. Nat Genet.

[53] Wu Y., Li X., Liu J., Luo X.J., Yao Y.G. (2020). SZDB2.0: An updated comprehensive resource for schizophrenia research. Hum Genet.

[54] Bipolar Disorder and Schizophrenia Working Group of the Psychiatric Genomics Consortium (2018). Genomic dissection of bipolar disorder and schizophrenia, including 28 subphenotypes. Cell.

[55] Deciphering Developmental Disorders Study (2017). Prevalence and architecture of de novo mutations in developmental disorders. Nature.

[56] Cotney J., Muhle R.A., Sanders S.J., Liu L., Willsey A.J., Niu W. (2015). The autism-associated chromatin modifier CHD8 regulates other autism risk genes during human neurodevelopment. Nat Commun.

[57] Matsuoka Y., Hughes C.A., Bennett V. (1996). Adducin regulation. Definition of the calmodulin-binding domain and sites of phosphorylation by protein kinases A and C. J Biol Chem.

[58] Matsuoka Y., Li X., Bennett V. (2000). Adducin: Structure, function and regulation. Cell Mol Life Sci.

[59] Jackman S.L., Turecek J., Belinsky J.E., Regehr W.G. (2016). The calcium sensor synaptotagmin 7 is required for synaptic facilitation. Nature.

[60] Buraei Z., Yang J. (2013). Structure and function of the β subunit of voltage-gated Ca^2+^ channels. Biochim Biophys Acta.

[61] Bando Y., Hirano T., Tagawa Y. (2014). Dysfunction of KCNK potassium channels impairs neuronal migration in the developing mouse cerebral cortex. Cereb Cortex.

[62] Torrico B., Shaw A.D., Mosca R., Vivó-Luque N., Hervás A., Fernàndez-Castillo N. (2019). Truncating variant burden in high-functioning autism and pleiotropic effects of LRP1 across psychiatric phenotypes. J Psychiatry Neurosci.

[63] Bosia M., Pigoni A., Zagato L., Merlino L., Casamassima N., Lorenzi C. (2016). ADDing a piece to the puzzle of cognition in schizophrenia. Eur J Med Genet.

[64] Juraeva D., Haenisch B., Zapatka M., Frank J., GROUP Investigators, PSYCH-GEMS SCZ Working Group (2014). Integrated pathway-based approach identifies association between genomic regions at CTCF and CACNB2 and schizophrenia. PLoS Genet.

[65] Shen W., Wang Q.W., Liu Y.N., Marchetto M.C., Linker S., Lu S.Y. (2020). Synaptotagmin-7 is a key factor for bipolar-like behavioral abnormalities in mice. Proc Natl Acad Sci U S A.

[66] Nguyen L.S., Lepleux M., Makhlouf M., Martin C., Fregeac J., Siquier-Pernet K. (2016). Profiling olfactory stem cells from living patients identifies miRNAs relevant for autism pathophysiology. Mol Autism.

[67] Bossy-Wetzel E., Schwarzenbacher R., Lipton S.A. (2004). Molecular pathways to neurodegeneration. Nat Med.

[68] Brennand K., Savas J.N., Kim Y., Tran N., Simone A., Hashimoto-Torii K. (2015). Phenotypic differences in hiPSC NPCs derived from patients with schizophrenia. Mol Psychiatry.

[69] Yu D.X., Di Giorgio F.P., Yao J., Marchetto M.C., Brennand K., Wright R. (2014). Modeling hippocampal neurogenesis using human pluripotent stem cells [published correction appears in Stem Cell Reports 2014; 3:217]. Stem Cell Reports.

[70] Sarkar A., Mei A., Paquola A.C.M., Stern S., Bardy C., Klug J.R. (2018). Efficient generation of CA3 neurons from human pluripotent stem cells enables modeling of hippocampal connectivity in vitro. Cell Stem Cell.

[71] Tang T.S., Slow E., Lupu V., Stavrovskaya I.G., Sugimori M., Llinás R. (2005). Disturbed Ca2+ signaling and apoptosis of medium spiny neurons in Huntington’s disease. Proc Natl Acad Sci U S A.

[72] Bezprozvanny I., Hayden M.R. (2004). Deranged neuronal calcium signaling and Huntington disease. Biochem Biophys Res Commun.

[73] Bezprozvanny I. (2009). Calcium signaling and neurodegenerative diseases. Trends Mol Med.

[74] Raymond L.A. (2017). Striatal synaptic dysfunction and altered calcium regulation in Huntington disease. Biochem Biophys Res Commun.

[75] Mackay J.P., Nassrallah W.B., Raymond L.A. (2018). Cause or compensation?-Altered neuronal Ca^2+^ handling in Huntington’s disease. CNS Neurosci Ther.

[76] Uematsu K., Heiman M., Zelenina M., Padovan J., Chait B.T., Aperia A. (2015). Protein kinase A directly phosphorylates metabotropic glutamate receptor 5 to modulate its function. J Neurochem.

[77] Bibb J.A., Yan Z., Svenningsson P., Snyder G.L., Pieribone V.A., Horiuchi A. (2000). Severe deficiencies in dopamine signaling in presymptomatic Huntington’s disease mice. Proc Natl Acad Sci U S A.

[78] Lin J.T., Chang W.C., Chen H.M., Lai H.L., Chen C.Y., Tao M.H., Chern Y. (2013). Regulation of feedback between protein kinase A and the proteasome system worsens Huntington’s disease. Mol Cell Biol.

[79] Yger M., Girault J.A. (2011). DARPP-32, jack of all trades . . . master of which?. Front Behav Neurosci.

[80] Kunii Y., Hyde T.M., Ye T., Li C., Kolachana B., Dickinson D. (2014). Revisiting DARPP-32 in postmortem human brain: Changes in schizophrenia and bipolar disorder and genetic associations with t-DARPP-32 expression. Mol Psychiatry.

[81] Albert K.A., Hemmings H.C., Adamo A.I.B., Potkin S.G., Akbarian S., Sandman C.A. (2002). Evidence for decreased DARPP-32 in the prefrontal cortex of patients with schizophrenia. Arch Gen Psychiatry.

[82] Peixoto R.T., Chantranupong L., Hakim R., Levasseur J., Wang W., Merchant T. (2019). Abnormal striatal development underlies the early onset of behavioral deficits in Shank3B^-/-^ mice. Cell Rep.

